# Problematic smartphone use in college students: the interplay of adverse childhood experiences and lifestyle factors

**DOI:** 10.3389/fpubh.2026.1940725

**Published:** 2026-08-31

**Authors:** Yanling Shu, Yunyun Liu, Wenhua Wang, Linfei Dou, Xinghua Yang, Xirennayi Abudurexiti, Lei Zhang, Mingyang Wu, Youjie Wang

**Affiliations:** 1 Department of Maternal and Child Health, School of Public Health, Tongji Medical College, Huazhong University of Science and Technology, Wuhan, Hubei, China; 2 Department of Preventive Medicine, School of Medical Technology, Jiangsu College of Nursing, Huai'an, Jiangsu, China; 3 Shaanxi Medical Association, Xi'an, China; 4 Shaanxi Provincial Health Industry Association Service Center, Xi'an, China; 5 Department of Maternal and Child Health, Xiangya School of Public Health, Central South University, Changsha, China

**Keywords:** adverse childhood experiences, college students, interplay, lifestyle, problematic smartphone use

## Abstract

**Background:**

The interplay between adverse childhood experiences (ACEs) and lifestyles in influencing problematic smartphone use (PSU) remains unclear. Therefore, we aimed to examine the associations between ACEs and PSU across different lifestyles among college students in China.

**Methods:**

The participants from six universities were randomly chosen from 57 universities in Shaanxi province in China, using a multistage, random cluster sampling method. ACEs and lifestyles were evaluated using a structured questionnaire and PSU was assessed using the Mobile Phone Addiction Tendency Scale. Binary logistic regression models and additive interaction models were employed to examine the associations between ACEs and PSU across different lifestyles among college students.

**Results:**

Among the 18,649 college students, 5,538 (29.70%) reported having PSU, and 82.59% of participants had encountered at least one ACE, with 10.69% having experienced four or more ACEs. Among college students with unhealthy lifestyles, students experiencing ≥ 4 ACEs (*OR*, 5.82; 95% *CI*, 4.30–7.92) or any of four ACE categories showed a markedly elevated likelihood of PSU. ACEs and unhealthy lifestyle exerted significant additive effects on the risk of PSU (relative excess risk due to the interaction [*RERI*]: 3.19 for ≥ 4 ACEs, 0.84 for household dysfunction, 1.23 for neglect; attributable proportion due to the interaction [*AP*]: 0.55 for ≥ 4 ACEs, 0.24 for household dysfunction, 0.34 for neglect).

**Conclusions:**

Our findings suggest that the ACEs and unhealthy lifestyles were associated with elevated likelihood of engaging in PSU among college students. Furthermore, there were positive additive interactions between ACEs and lifestyle factors in relation to PSU.

## 1 Introduction

In the digital age, smartphones have become ubiquitous, profoundly influencing daily life, especially among college students. These young adults are characterized by high smartphone usage, driven by course learning, academic demands, social interactions, shopping consumption, and entertainment (1, 2). This pervasive use, however, raises concerns about problematic smartphone use (PSU), a growing phenomenon marked by excessive and compulsive use of smartphones. Previous research has indicated that PSU is related to a range of negative outcomes (3, 4), including diminished psychological wellbeing (5), impaired social relationships (6), and reduced academic performance (7, 8). As mobile technology increasingly integrates into the daily lives of college students, understanding the underlying factors contributing to PSU is essential for formulating effective prevention and intervention strategies.

Recent investigations have emphasized that adverse childhood experiences (ACEs), defined as potentially traumatic events occurring before the age of 18, are well-established risk factors for various psychological and behavioral problems in adulthood (9–11). Individuals with a history of ACEs frequently resort to maladaptive coping strategies, which may heighten their susceptibility to addictive behaviors (12). Several studies have demonstrated that young adults who have encountered ACEs have a higher risk of engaging in problematic gaming (13), excessive internet use (14) and substance misuse (12). Nevertheless, few studies have explored the association between ACEs and PSU with a large representative sample. Furthermore, lifestyle choices, including patterns of physical activity, smoking, alcohol drinking, and diet habits, play a critical role in shaping individuals' vulnerability to PSU. Prior studies suggest that regular physical activity and a healthy diet may serve as protective factors against PSU (15, 16). However, the interaction between ACEs and lifestyle choices in influencing PSU has not yet been comprehensively examined and remains an area of significant interest. Understanding this interaction could provide valuable insights for targeted preventive measures and therapeutic approaches.

Therefore, in the present study, we examine the associations of ACEs and lifestyle factors with PSU among college students across six universities in China. By employing a comprehensive approach that integrates both direct and interactive effects of ACEs and lifestyles, we aim to elucidate how these elements collectively contribute to the development of PSU. We hypothesize that unhealthy lifestyle choices may exacerbate the effects of ACEs on PSU, creating a compounded risk for PSU. This research will enhance the understanding of the underlying risk factors for PSU and guide the development of targeted interventions that address both historical and behavioral contributors. Through this exploration, we seek to advance the field's knowledge and provide evidence-based recommendations for mitigating the impact of PSU on young adults.

## 2 Methods

### 2.1 Study design, data collection, and participants

This cross-sectional survey was carried out in Shaanxi Province, China, between October and November 2022, and adopted a rigorous multistage stratified cluster sampling design. In the first sampling stage, stratified random sampling was conducted according to institutional attributes: four public undergraduate universities were randomly selected from 34 local public universities, and two private undergraduate universities were randomly selected from 23 local private universities in Shaanxi Province. All sampled institutions were regular comprehensive undergraduate universities without vocational colleges included. In the second stage, systematic cluster sampling was adopted, with 2–4 classes randomly selected from each grade and each discipline of every included university. A unified sampling proportion of 6.25% was strictly implemented across all six universities, and all students in the selected 559 classes were recruited, yielding an initial sample of 20,165 participants.

Before data collection, two class representatives from each participating class received standardized training. These representatives then facilitated the questionnaire completion by instructing other students to use a quick response code (QR code) for access, thereby improving both accuracy and efficiency. To ensure data quality, the questionnaire included a basic arithmetic question (e.g., solving 2 multiplied by 12, then by 3) and a categorical question (e.g., identifying which of the following is not an animal: rose flower, monkey, panda, or cow). Out of the 19,622 students who completed the online questionnaire, responses were excluded for incomplete submissions, completion times shorter than 500 seconds (based on pretesting and 1st percentile cut-off), incorrect answers to quality control questions, and missing lifestyle data. Consequently, the final analysis included 18,649 students. Electronic informed consent was provided by all participants.

### 2.2 Assessment of adverse childhood experiences (ACEs)

ACEs were assessed using the Adverse Childhood Experiences International Questionnaire (ACE-IQ), a tool created by the World Health Organization (WHO). This questionnaire evaluates 13 distinct domains of adverse experiences, which encompass various forms of household dysfunction (substance abuse, incarcerated family member, mental illness, family violence, parental separation or death), child neglect (physical, emotional), child abuse (physical, emotional, sexual), bullying, community violence, and collective violence. Each item in the questionnaire offers two response options: “Ever” and “Never,” scored as 1 for “Ever” and 0 for “Never.” In this research, the ACE-IQ demonstrated a Cronbach's alpha reliability coefficient of 0.69. The overall ACE score was calculated by summing the responses from all 13 items (with possible scores ranging from 0 to 13), indicating the level of ACE exposure. Participants were subsequently categorized into five groups based on their total ACE scores: 0, 1, 2, 3, and four or more (17).

### 2.3 Assessment of lifestyle factors

Lifestyle factors were assessed using a structured questionnaire. Unhealthy lifestyle factors were defined as alcohol drinking, current smoking, inadequate physical activity, unhealthy diet, and abnormal weight (obesity or underweight) (18, 19). Current smokers were defined as individuals who had smoked at least one cigarette within the last 30 days, while those classified as current alcohol drinkers had consumed at least one alcoholic beverage in the same timeframe. Physical activity levels were evaluated using the International Physical Activity Questionnaire Short Form (IPAQ-SF), which categorized participants into low, moderate, and high activity levels based on metabolic equivalents (METs) (20). High level of physical activity was indicated by either (a) ≥3 days of vigorous intensity activity and ≥ 1,500 MET min per week, or (b) ≥7 days of any combination of walking, moderate or vigorous intensity activities and ≥ 3,000 MET min per week. Moderate level of activity was defined as (a) ≥3 days of vigorous intensity activity for at least 20 min per day, (b) ≥5 days of moderate intensity activity and/or walking of at least 30 min per day, or (c) ≥5 days of any combination of walking, moderate or vigorous intensity activities and ≥ 600 MET min per week. Participants not meeting the criteria for either “moderate” or “high” level of physical activity were classified as having low level of physical activity, which was deemed unhealthy for this study. Body mass index (BMI) was calculated by dividing weight by the square of height, with underweight defined as BMI < 18.5 kg/m^2^ and obesity as BMI > 28 kg/m^2^. An unhealthy diet was characterized by daily red meat consumption or fewer than daily servings of vegetables/fruits (19, 21). Each unhealthy behavior received a score of one, and the total score across five behaviors formed the lifestyle risk index, ranging from 0 to 5. Participants were classified into three categories based on their lifestyle risk index: healthy lifestyle ( ≤ 1), intermediate lifestyle (2), and unhealthy lifestyle (≥3), in line with prior research (22, 23).

### 2.4 Assessment of problematic smartphone use (PSU)

PSU was assessed by the Mobile Phone Addiction Tendency Scale (MPATS), which was designed by Xiong et al. (24) specifically for Chinese college students. This scale comprises 16 items divided into four dimensions: salience, social comfort, withdrawal symptoms, and mood changes. Each item is rated on a scale from 1 (very inconsistent) to 5 (very consistent), yielding a total score that ranges from 16 to 80. Higher scores on the scale reflect greater levels of PSU, with scores of 48 or above being indicative of PSU. In this study, the scale demonstrated strong internal consistency, indicated by a Cronbach's alpha coefficient of 0.94.

### 2.5 Covariate assessment

Covariates including gender (male or female), race (Han or others), place of residence (rural or urban), grade (1st, 2nd, 3rd, 4th or above), sibship status (only child or having siblings), family structure (two-parent family or others), parental education (middle school or below, high school, college or above) were obtained through a custom-designed general information questionnaire.

### 2.6 Statistical analysis

All analyses were performed using R version 4.0.2. Categorical variables were reported as counts with percentages and analyzed using Chi-squared tests. Continuous variables were presented as means with standard deviations (SD) for normally distributed data, and compared using *t*-tests or analysis of variance (ANOVA). For data exhibiting skewed distributions, medians (25th−75th percentiles) were reported and comparisons were made using the Wilcoxon signed-rank test or Kruskal-Wallis H test. Logistic regression models were employed to evaluate the relationships between ACEs and lifestyles with PSU. Three models were utilized: Model 1 served as the crude model; Model 2 controlled for gender, race, place of residence and grade; and Model 3 additionally adjusted for sibship status, family structure, maternal education and paternal education. In Model 3, lifestyle factors were also adjusted for in the relationship between ACEs and PSU, and ACE scores were adjusted in the association between lifestyles and PSU. To eliminate statistical bias caused by nested cluster sampling structure, multilevel binary logistic regression models with random intercepts for classes nested within schools were also applied to evaluate the associations of ACEs and lifestyle with PSU. Furthermore, the joint effects of ACEs and lifestyle on PSU were also explored using additive interaction models. Interaction effects were assessed by two indexes: the attributable proportion (*AP*) of the interaction and the relative excess risk due to interaction (*RERI*) (25). Confidence intervals (*CIs*) for *AP* and *RERI* were calculated using 5,000 bootstrap samples (26); an absence of significant additive interaction was indicated if the *CIs* for *AP* and *RERI* included zero.

## 3 Results

### 3.1 Participant characteristics

Table 1 details the characteristics of the college students categorized by their engagement in PSU, and the characteristics categorized by lifestyle factors were provided in Supplementary Table S1. Among the 18,649 college students, 12,137 (65.08%) were female, and 5,538 (29.70%) reported having PSU, with the mean (SD) MPATS score of 39.51 (SD = 13.03). Of these, 82.59% of participants had encountered at least one ACE, with 10.69% having faced four or more ACEs. Participants with PSU were more likely to be in lower academic years, adopt unhealthy lifestyles, and report higher ACE exposure (Table 1). Furthermore, the PSU group also showed higher frequencies of abuse, household dysfunction, neglect, and violence. Additionally, participants with unhealthy lifestyles included a higher proportion of male students and reported greater exposure to ACEs (Supplementary Table S1).

**Table 1:** Characteristics of study participants according to problematic smartphone use (PSU).

Characteristics	Overall (*N* = 18,649)	Non-PSU (*N* = 13,111)	PSU (*N* = 5,538)	*P*
Gender, *n* (%)				0.054
Male	6,512 (34.92)	4,636 (35.36)	1,876 (33.88)	
Female	12,137 (65.08)	8,475 (64.64)	3,662 (66.12)	
Grade, *n* (%)				< 0.001
1st	5,436 (29.15)	3,903 (29.77)	1,533 (27.68)	
2nd	4,448 (23.85)	2,955 (22.54)	1,493 (26.96)	
3rd	4,379 (23.48)	3,065 (23.38)	1,314 (23.73)	
4th+	4,386 (23.52)	3,188 (24.32)	1,198 (21.63)	
Race, *n* (%)				0.008
Han	18,102 (97.07)	12,755 (97.28)	5,347 (96.55)	
Others	547 (2.93)	356 (2.72)	191 (3.45)	
Place of residence, *n* (%)				0.964
Rural	10,065 (53.97)	7,078 (53.99)	2,987 (53.94)	
Urban	8,584 (46.03)	6,033 (46.01)	2,551 (46.06)	
Sibship, *n* (%)				0.406
Only child	5,513 (29.56)	3,900 (29.75)	1,613 (29.13)	
Having sibling(s)	13,136 (70.44)	9,211 (70.25)	3,925 (70.87)	
Family structure				0.045
Two-parent family	16,338 (87.61)	11,528 (87.93)	4,810 (86.85)	
Others	2,311 (12.39)	1,583 (12.07)	728 (13.15)	
Maternal education, *n* (%)				0.645
Middle school or under	11,987 (64.28)	8,400 (64.07)	3,587 (64.77)	
High school	3,764 (20.18)	2,658 (20.27)	1,106 (19.97)	
College or above	2,898 (15.54)	2,053 (15.66)	845 (15.26)	
Paternal education, *n* (%)				0.258
Middle school or under	10,188 (54.63)	7,125 (54.34)	3,063 (55.31)	
High school	4,114 (22.06)	2,934 (22.38)	1,180 (21.31)	
College or above	4,347 (23.31)	3,052 (23.28)	1,295 (23.38)	
Lifestyles, *n* (%)				< 0.001
Healthy	2,762 (14.81)	2,168 (16.54)	594 (10.73)	
Intermediate	13,973 (74.93)	9,749 (74.36)	4,224 (76.27)	
Unhealthy	1,914 (10.26)	1,194 (9.11)	720 (13.00)	
Number of ACEs, *n* (%)				< 0.001
0	3,246 (17.41)	2,626 (20.03)	620 (11.20)	
1	10,195 (54.67)	7,168 (54.67)	3,027 (54.66)	
2	2,054 (11.01)	1,390 (10.60)	664 (11.99)	
3	1,160 (6.22)	747 (5.70)	413 (7.46)	
≥4	1,994 (10.69)	1,180 (9.00)	814 (14.70)	
Abuse, *n* (%)				< 0.001
No	14,454 (77.51)	10,418 (79.46)	4,036 (72.88)	
Yes	4,195 (22.49)	2,693 (20.54)	1,502 (27.12)	
Household dysfunction, *n* (%)				< 0.001
No	15,110 (81.02)	10,802 (82.39)	4,308 (77.79)	
Yes	3,539 (18.98)	2,309 (17.61)	1,230 (22.21)	
Neglect, *n* (%)				< 0.001
No	6,596 (35.37)	5,074 (38.70)	1,522 (27.48)	
Yes	12,053 (64.63)	8,037 (61.30)	4,016 (72.52)	
Violence, *n* (%)				< 0.001
No	15,072 (80.82)	10,760 (82.07)	4,312 (77.86)	
Yes	3,577 (19.18)	2,351 (17.93)	1,226 (22.14)	
MPATS score, mean (SD)	39.51(13.03)	33.14(9.12)	54.58(7.21)	< 0.001
ACE score, median (IQR)	1.00(1.00–2.00)	1.00(1.00–2.00)	1.00(1.00–2.00)	< 0.001

PSU, problematic smartphone use; ACEs, adverse childhood experiences; MPATS, Mobile Phone Addiction Tendency Scale; SD, standard deviation; IQR, inter-quartile range.

### 3.2 Associations between ACEs and PSU

Table 2 shows that a higher ACE score among college students was related to an increased likelihood of PSU. Compared to individuals who did not experience ACEs, students with at least one ACE showed an increased likelihood of PSU (e.g., ≥4 ACEs: *OR*, 2.86; 95% *CI*, 2.52–3.26). Specifically, students who experienced abuse, household dysfunction, neglect and violence exhibited a significantly increased likelihood of PSU compared to those without such experiences (Abuse: *OR*, 1.41; 95% *CI*, 1.31–1.52; Household dysfunction: *OR*, 1.35; 95% *CI*, 1.24–1.48; Neglect: *OR*, 1.65; 95% *CI*, 1.54–1.77; Violence: *OR*, 1.28; 95% *CI*, 1.18–1.38) (Table 2). Additionally, the core significant associations between ACEs and PSU remained robust in the multilevel binary logistic regression model (Supplementary Table S2).

**Table 2:** Associations between adverse childhood experiences (ACEs) and problematic smartphone use (PSU) among study participants.

ACEs	***OR*** **(95%** ***CI*****)**		
	Model 1	Model 2	Model 3
ACE score	1.13 (1.11–1.16)	1.14 (1.12–1.16)	**1.13 (1.11–1.15)**
Abuse	1.44 (1.34–1.55)	1.45 (1.34–1.56)	**1.41 (1.31–1.52)**
Physical	1.41 (1.31–1.52)	1.42 (1.31–1.53)	**1.38 (1.28–1.49)**
Emotional	1.55 (1.42–1.69)	1.56 (1.42–1.70)	**1.51 (1.38–1.65)**
Sexual	1.41 (1.15–1.73)	1.40 (1.14–1.71)	**1.37 (1.11–1.68)**
Household dysfunction	1.34 (1.24–1.44)	1.33 (1.23–1.44)	**1.35 (1.24–1.48)**
Substance abuse	1.43 (1.13–1.80)	1.42 (1.12–1.79)	**1.35 (1.06–1.70)**
Incarcerated household member	1.20 (0.95–1.52)	1.21 (0.95–1.52)	1.13 (0.89–1.43)
Mental illness	1.38 (1.15–1.64)	1.37 (1.15–1.63)	**1.30 (1.09–1.55)**
Family violence	1.59 (1.43–1.76)	1.59 (1.43–1.76)	**1.54 (1.39–1.71)**
Parental death or separation	1.13 (1.03–1.25)	1.12 (1.02–1.24)	1.10 (0.96–1.26)
Neglect	1.67 (1.56–1.78)	1.68 (1.56–1.80)	**1.65 (1.54–1.77)**
Emotional	1.65 (1.54–1.77)	1.66 (1.55–1.77)	**1.63 (1.52–1.75)**
Physical	1.33 (1.05–1.67)	1.33 (1.05–1.67)	**1.30 (1.03–1.64)**
Violence	1.30 (1.20–1.41)	1.31 (1.21–1.42)	**1.28 (1.18–1.38)**
Bullying	1.54 (1.40–1.70)	1.56 (1.41–1.71)	**1.52 (1.38–1.67)**
Community violence	1.21 (1.10–1.32)	1.22 (1.11–1.33)	**1.18 (1.08–1.29)**
Collective violence	1.39 (1.10–1.75)	1.39 (1.10–1.74)	**1.32 (1.05–1.66)**
**Number of ACEs**			
0	Ref.	Ref.	Ref.
1	1.79 (1.62–1.97)	1.81 (1.64–1.99)	**1.77 (1.61–1.96)**
2	2.02 (1.78–2.30)	2.04 (1.79–2.32)	**2.02 (1.78–2.30)**
3	2.34 (2.02–2.72)	2.37 (2.04–2.75)	**2.30 (1.98–2.68)**
≥4	2.92 (2.58–3.31)	2.96 (2.61–3.36)	**2.86 (2.52–3.26)**

*OR*, odds ratio; *CI*, confidence interval; Model 1 was crude model; Model 2 was further adjusted for gender, race, place of residence and grade; Model 3 was additionally adjusted for sibship, family structure, maternal education, paternal education, and lifestyles. Bold values represent statistically significant results (*P* < 0.05).

### 3.3 Associations between lifestyles and PSU

The relationships between lifestyle factors and PSU are presented in Table 3. After controlling for confounding variables, the association between lifestyle factors and PSU remained significant. Compared to students with a healthy lifestyle, the likelihood of PSU was higher (*OR*, 1.56; 95% *CI*, 1.42–1.73) for students with an intermediate lifestyle or higher (*OR*, 2.15; 95% *CI*, 1.88–2.46) for students with an unhealthy lifestyle. Moreover, in multilevel binary logistic regression model, the significant associations between lifestyles and PSU were not changed materially (Supplementary Table S3).

**Table 3:** Associations between lifestyles and problematic smartphone use (PSU) among study participants.

Lifestyles	***OR*** **(95%** ***CI*****)**		
	Model 1	Model 2	Model 3
Healthy	Ref.	Ref.	Ref.
Intermediate	1.58 (1.44–1.74)	1.59 (1.44–1.75)	**1.56 (1.42–1.73)**
Unhealthy	2.20 (1.93–2.51)	2.30 (2.01–2.62)	**2.15 (1.88–2.46)**

*OR*, odds ratio; *CI*, confidence interval; Model 1 was crude model; Model 2 was further adjusted for gender, race, place of residence and grade; Model 3 was additionally adjusted for sibship, family structure, maternal education, paternal education, and ACE score. Bold values represent statistically significant results (*P* < 0.05).

### 3.4 Joint effect of ACEs and lifestyles on PSU

Table 4 illustrates the joint effects of cumulative exposure to ACEs and lifestyle factors on PSU. College students who reported experiencing at least one ACE and who had intermediate or unhealthy lifestyle were found to have an increased likelihood of PSU. Specifically, among those with intermediate lifestyle, students who experienced four or more ACEs showed a markedly higher probability of engaging in PSU (*OR*, 3.67; 95% *CI*, 2.90–4.69). Similarly, students with an unhealthy lifestyle and four or more ACEs showed a markedly elevated likelihood of PSU (*OR*, 5.82; 95% *CI*, 4.30–7.92). The interaction analysis revealed positive additive effects between the number of ACEs and lifestyles in relation to PSU. Compared to the students with no ACEs and a healthy lifestyle, those with four or more ACEs and an unhealthy lifestyle had a higher likelihood of PSU (*RERI*, 3.19, 95% *CI*, 1.61–4.76); the ACE-lifestyle interaction was responsible for 55% of the PSU among students with four or more ACEs and an unhealthy lifestyle (*AP*, 0.55, 95% *CI*, 0.37–0.72).

**Table 4:** Joint effect of the number of adverse childhood experiences (ACEs) and lifestyles on problematic smartphone use (PSU).

Number of ACEs	Lifestyles						
	*OR* (95%*CI*)			*RERI* (95%*CI*)		*AP* (95%*CI*)	
	Healthy	Intermediate	Unhealthy	Intermediate	Unhealthy	Intermediate	Unhealthy
0	Ref.	1.35 (1.07–1.72)	0.99 (0.60–1.57)				
1	1.40 (1.10–1.81)	2.38 (1.92–2.99)	3.35 (2.62–4.33)	0.63 (0.37–0.90)	**1.96 (1.30–2.62)**	0.26 (0.13–0.40)	**0.59 (0.43–0.74)**
2	1.94 (1.38–2.72)	2.69 (2.12–3.44)	3.38 (2.40–4.75)	0.40 (−0.22–1.01)	**1.44 (0.33–2.56)**	0.15 (−0.08–0.38)	**0.43 (0.17–0.68)**
3	2.16 (1.40–3.31)	3.07 (2.38–3.97)	3.96 (2.68–5.86)	0.55 (−0.37–1.48)	**1.81 (0.22–3.41)**	0.18 (−0.12–0.48)	**0.46 (0.16–0.75)**
≥4	2.65 (1.87–3.74)	3.67 (2.90–4.69)	5.82 (4.30–7.92)	0.68 (−0.15–1.51)	**3.19 (1.61–4.76)**	0.18 (−0.04–0.41)	**0.55 (0.37–0.72)**

*OR*, odds ratio; *CI*, confidence interval; *RERI*, relative excess risk due to interaction; *AP*, attributable proportion due to interaction; Model was adjusted for gender, race, place of residence, grade, sibship, family structure, maternal education, and paternal education. Bold values represent statistically significant results (*P* < 0.05).

Table 5 demonstrates that experiencing any of the four ACE categories (abuse, household dysfunction, neglect, and violence) correlated with increased likelihood of PSU across different lifestyle groups. Among college students with an unhealthy lifestyle, those with a history of abuse showed a significantly increased likelihood of PSU (*OR*, 3.22; 95% *CI*, 2.64–3.91), followed by household dysfunction (*OR*, 3.52; 95% *CI*, 2.84–4.35), neglect (*OR*, 3.64; 95% *CI*, 3.02–4.40) and violence (*OR*, 2.95; 95% *CI*, 2.41–3.61). Furthermore, the results suggested that there was a potential synergistic effect between household dysfunction or neglect and an unhealthy lifestyle on PSU (Household dysfunction: *RERI*, 0.84; 95% *CI*, 0.09–1.59; *AP*, 0.24; 95% *CI*, 0.06–0.42; Neglect: *RERI*, 1.23; 95% *CI*, 0.67–1.79; *AP*, 0.34; 95% *CI*, 0.20–0.47).

**Table 5:** Joint effect of categorized adverse childhood experiences (ACEs) and lifestyles on problematic smartphone use (PSU).

ACE category	Lifestyles						
	*OR* (95%*CI*)			*RERI* (95%*CI*)		*AP* (95%*CI*)	
	Healthy	Intermediate	Unhealthy	Intermediate	Unhealthy	Intermediate	Unhealthy
Abuse	
ACE not reported	1 (Ref)	1.61 (1.44–1.80)	2.26 (1.93–2.64)				
ACE reported	1.52 (1.23–1.89)	2.24 (1.97–2.55)	3.22 (2.64–3.91)	0.11 (−0.24–0.46)	0.43 (−0.22–1.09)	0.05 (−0.11–0.20)	0.14 (−0.05–0.32)
Household dysfunction	
ACE not reported	1 (Ref)	1.62 (1.46–1.81)	2.19 (1.88–2.55)				
ACE reported	1.49 (1.18–1.87)	2.10 (1.83–2.41)	3.52 (2.84–4.35)	−0.01 (−0.38–0.36)	**0.84 (0.09–1.59)**	−0.01 (−0.18–0.17)	**0.24 (0.06–0.42)**
Neglect	
ACE not reported	1 (Ref)	1.50 (1.27–1.77)	1.85 (1.45–2.36)				
ACE reported	1.56 (1.29–1.88)	2.44 (2.08–2.86)	3.64 (3.02–4.40)	0.38 (0.14–0.63)	**1.23 (0.67–1.79)**	0.16 (0.05–0.27)	**0.34 (0.20–0.47)**
Violence	
ACE not reported	1 (Ref)	1.62 (1.45–1.81)	2.28 (1.96–2.66)				
ACE reported	1.41 (1.12–1.76)	2.03 (1.78–2.32)	2.95 (2.41–3.61)	0.01 (−0.34–0.36)	0.26 (−0.38–0.90)	0.00 (−0.17–0.17)	0.09 (−0.12–0.30)

*OR*, odds ratio; *CI*, confidence interval; *RERI*, relative excess risk due to interaction; *AP*, attributable proportion due to interaction; Model was adjusted for gender, race, place of residence, grade, sibship, family structure, maternal education, and paternal education. Bold values represent statistically significant results (*P* < 0.05).

## 4 Discussion

This study is one of the first to investigate the relationships between ACEs and PSU according to different lifestyles among college students. The findings of the present study demonstrate that students subjected to ACEs or exhibiting unhealthy lifestyle patterns are at a significantly higher likelihood of PSU. Moreover, our results reveal positive additive interactions between the ACEs and lifestyle factors in relation to PSU. This additive effect suggests that the impact of ACEs on PSU is not simply cumulative but is exacerbated by unhealthy lifestyle choices.

The present study indicates that college students with a history of ACEs were more likely to engage in unhealthy lifestyles, which is consistent with existing research (27). Given the chronic stress associated with ACEs (28), these individuals may gravitate toward socially patterned unhealthy behaviors (e.g., poor diet, smoking, or drinking) as a means of stress avoidance or reduction (29). Additionally, our findings highlight a significant correlation between higher ACE scores and increased propensity for PSU. Specifically, students with backgrounds of abuse, household dysfunction, neglect, and violence exhibit significantly higher tendencies toward PSU compared to those without such adverse experiences. These results align with limited studies conducted in China and the United States of America (30, 31). The heightened vulnerability to PSU among those with a traumatic past may be explained by several psychological mechanisms. For instance, individuals with a history of ACEs might use smartphones as a coping strategy or escape from stressors (32, 33). Furthermore, early ACEs may lead to enduring alterations in dopamine, oxytocin, and glucocorticoid systems, which could be linked to addiction at molecular, neuroendocrine, and behavioral levels (34).

Additionally, our study underscores a significant association between unhealthy lifestyle behaviors and an increased susceptibility to PSU among college students. This finding aligns with earlier studies suggesting that lifestyle factors such as physical inactivity and sedentary behavior are associated with PSU (15, 35). This association may be partly explained by several pathways. For instance, unhealthy lifestyles, such as unhealthy dietary habits and lack of physical activity (36, 37), can contribute to increased stress and anxiety levels. These psychological states may, in turn, drive individuals to seek refuge in smartphone use as a coping mechanism (38). Moreover, unhealthy lifestyle behaviors can lead to diminished self-regulation and time management skills (39, 40), which are crucial for controlling smartphone usage.

Furthermore, we also observed notable joint effects of ACEs and lifestyles on PSU. Specifically, a greater prevalence of PSU was found when a higher number of ACEs were combined with unhealthy lifestyle. A potential synergistic effect between household dysfunction or neglect and unhealthy lifestyle factors on PSU was also observed. This finding implies that the combination of household dysfunction or neglect during childhood and concurrent unhealthy lifestyle may create a particularly strong predisposition toward PSU. Household dysfunction or neglect may lead to emotional and psychological vulnerabilities, which are then aggravated by unhealthy lifestyles such as lack of physical activity, smoking, and unhealthy diet habits. Currently, research has not extensively investigated the relationship between ACEs and PSU across various lifestyles. However, existing research has revealed that ACEs are related to other mental health and behavioral problems across different lifestyles. For instance, studies conducted in China have shown that college students who have higher ACE scores and unhealthy lifestyles are at an increased risk of engaging in nonsuicidal self-injury (21). The findings of our study underscore the importance of addressing both early-life adversities and lifestyle factors in prevention and intervention efforts for PSU. Strategies aimed at reducing PSU should incorporate components that address both the psychological impacts of ACEs and the promotion of healthy lifestyle habits.

## 5 Limitations

This study has several limitations. Firstly, the cross-sectional design limits our capacity to draw causal inferences regarding the association between ACEs and PSU among college students with different lifestyles. Secondly, as our research focused exclusively on Chinese college students, the applicability of our findings to other age groups may be constrained. Thirdly, self-reported measures of PSU, ACEs, and lifestyle factors may introduce reporting biases. Fourthly, although the models adjusted for most individual and parental demographic variables, residual or unmeasured confounding variables may still exist and could potentially affect the results of our study. Future research would benefit from longitudinal studies to examine the causal relationships and temporal dynamics between ACEs, lifestyle factors, and PSU. Additionally, employing objective measures and incorporating a broader range of variables, such as social support systems and coping strategies, could enhance our understanding of these interactions.

## 6 Conclusions

In this cross-sectional study involving a representative sample of school-based participants, we observed that college students who reported experiencing ACEs were more likely to adopt unhealthy lifestyles. Students with unhealthy lifestyles or who were exposed to ACEs showed an increased vulnerability to PSU. Furthermore, the interplay between ACEs and unhealthy lifestyles further escalates the likelihood of PSU. Consequently, these findings suggest that mitigating the long-term psychological impacts of prior ACE exposure and promoting healthy lifestyle behaviors may reduce PSU risk among college students. At the population level, these results also underscore the importance of primary prevention of ACEs in childhood, which would reduce the downstream burden of behavioral problems in young adulthood.

## Data Availability

The datasets presented in this article are not readily available because data will be made available on request. Requests to access the datasets should be directed to shushu@hust.edu.cn.
