# Supplementary material for: Problematic smartphone use in college students: the interplay of adverse childhood experiences and lifestyle factors
Source: Front Public Health. 2026 Aug 31;14:1940725. doi: 10.3389/fpubh.2026.1940725 (PMC13572271; doi:10.3389/fpubh.2026.1940725)
Supplement: Supplementary file 1 [file Table_1.docx]

***Supplementary Table***

**Table S1.** **Characteristics of study participants** **across lifestyle categories.**

| Characteristics | Lifestyle | | | *P* |
| --- | --- | --- | --- | --- |
|  | Healthy | Intermediate | Unhealthy |  |
| Gender, n (%) |  |  |  | <0.001 |
| Male | 798 (28.89) | 4,551 (32.57) | 1,163 (60.76) |  |
| Female | 1,964 (71.11) | 9,422 (67.43) | 751 (39.24) |  |
| Grade, n (%) |  |  |  | <0.001 |
| 1st | 1,011 (36.60) | 3,902 (27.93) | 523 (27.32) |  |
| 2nd | 615 (22.27) | 3,377 (24.17) | 456 (23.82) |  |
| 3rd | 593 (21.47) | 3,315 (23.72) | 471 (24.61) |  |
| 4th+ | 543 (19.66) | 3,379 (24.18) | 464 (24.24) |  |
| Race, n (%) |  |  |  | 0.518 |
| Han | 2,690 (97.39) | 13,553 (96.99) | 1,859 (97.13) |  |
| Others | 72 (2.61) | 420 (3.01) | 55 (2.87) |  |
| Place of residence, n (%) |  |  |  | <0.001 |
| Rural | 1,514 (54.82) | 7,683 (54.98) | 868 (45.35) |  |
| Urban | 1,248 (45.18) | 6,290 (45.02) | 1,046 (54.65) |  |
| Sibship, n (%) |  |  |  | <0.001 |
| Only child | 814 (29.47) | 4,006 (28.67) | 693 (36.21) |  |
| Having sibling(s) | 1,948 (70.53) | 9,967 (71.33) | 1,221 (63.79) |  |
| Family structure, n (%) |  |  |  | 0.013 |
| Two-parent family | 2,449 (88.67) | 12,247 (87.65) | 1,642 (85.79) |  |
| Others | 313 (11.33) | 1,726 (12.35) | 272 (14.21) |  |
| Maternal education, n (%) |  |  |  | <0.001 |
| Middle school or under | 1,781 (64.48) | 9,066 (64.88) | 1,140 (59.56) |  |
| High school | 561 (20.31) | 2,800 (20.04) | 403 (21.06) |  |
| College or above | 420 (15.21) | 2,107 (15.08) | 371 (19.38) |  |
| Paternal education, n (%) |  |  |  | 0.132 |
| Middle school or under | 1,499 (54.27) | 7,693 (55.06) | 996 (52.04) |  |
| High school | 620 (22.45) | 3,059 (21.89) | 435 (22.73) |  |
| College or above | 643 (23.28) | 3,221 (23.05) | 483 (25.24) |  |
| PSU, n (%) |  |  |  | <0.001 |
| No | 2,168 (78.49) | 9,749 (69.77) | 1,194 (62.38) |  |
| Yes | 594 (21.51) | 4,224 (30.23) | 720 (37.62) |  |
| Number of ACEs, n (%) |  |  |  | <0.001 |
| 0 | 634 (22.95) | 2,444 (17.49) | 168 (8.78) |  |
| 1 | 1,457 (52.75) | 7,720 (55.25) | 1,018 (53.19) |  |
| 2 | 293 (10.61) | 1,522 (10.89) | 239 (12.49) |  |
| 3 | 135 (4.89) | 873 (6.25) | 152 (7.94) |  |
| ≥4 | 243 (8.80) | 1,414 (10.12) | 337 (17.61) |  |
| Abuse, n (%) |  |  |  | <0.001 |
| No | 2,211 (80.05) | 10,908 (78.06) | 1,335 (69.75) |  |
| Yes | 551 (19.95) | 3,065 (21.94) | 579 (30.25) |  |
| Household dysfunction, n (%) |  |  |  | <0.001 |
| No | 2,289 (82.87) | 11,382 (81.46) | 1,439 (75.18) |  |
| Yes | 473 (17.13) | 2,591 (18.54) | 475 (24.82) |  |
| Neglect, n (%) |  |  |  | <0.001 |
| No | 1,156 (41.85) | 4,895 (35.03) | 545 (28.47) |  |
| Yes | 1,606 (58.15) | 9,078 (64.97) | 1,369 (71.53) |  |
| Violence, n (%) |  |  |  | <0.001 |
| No | 2,274 (82.33) | 11,412 (81.67) | 1,386 (72.41) |  |
| Yes | 488 (17.67) | 2,561 (18.33) | 528 (27.59) |  |
| MPATS score, mean (SD) | 36.66(12.31) | 39.70(12.98) | 42.17(13.68) | <0.001 |
| ACEs score, median (IQR) | 1.00(1.00-1.00) | 1.00(1.00-2.00) | 1.00(1.00-3.00) | <0.001 |

**Note:** PSU, problematic smartphone use; MPATS, Mobile Phone Addiction Tendency Scale; ACEs, adverse childhood experiences; SD, standard deviation; IQR, inter-quartile range.

**Table S2. Associations between adverse childhood experiences (ACEs) and** **problematic smartphone use (PSU) among study participants (performed by multilevel model)**

| ACEs | *OR* (95%*CI*) | | |
| --- | --- | --- | --- |
|  | Model 1 | Model 2 | Model 3 |
| ACEs score | 1.13 (1.11-1.15) | 1.13 (1.11-1.15) | **1.12 (1.10-1.15)** |
| Abuse | 1.43 (1.32-1.54) | 1.43 (1.33-1.54) | **1.40 (1.30-1.51)** |
| Physical | 1.40 (1.29-1.51) | 1.40 (1.30-1.51) | **1.37 (1.26-1.48)** |
| Emotional | 1.53 (1.40-1.67) | 1.54 (1.40-1.68) | **1.49 (1.36-1.63)** |
| Sexual | 1.34 (1.09-1.64) | 1.33 (1.09-1.64) | **1.30 (1.06-1.60)** |
| Household dysfunction | 1.33 (1.23-1.44) | 1.33 (1.23-1.44) | **1.35 (1.23-1.48)** |
| Substance abuse | 1.38 (1.09-1.74) | 1.37 (1.08-1.74) | **1.30 (1.03-1.65)** |
| Incarcerated household member | 1.18 (0.93-1.50) | 1.18 (0.93-1.50) | 1.11 (0.87-1.41) |
| Mental illness | 1.35 (1.13-1.61) | 1.34 (1.12-1.60) | **1.28 (1.07-1.53)** |
| Family violence | 1.57 (1.42-1.75) | 1.57 (1.41-1.74) | **1.52 (1.37-1.70)** |
| Parental separation or divorce | 1.13 (1.03-1.25) | 1.13 (1.02-1.24) | 1.10 (0.96-1.26) |
| Neglect | 1.63 (1.52-1.75) | 1.64 (1.53-1.76) | **1.62 (1.51-1.74)** |
| Emotional | 1.61 (1.51-1.73) | 1.62 (1.51-1.74) | **1.60 (1.50-1.72)** |
| Physical | 1.28 (1.01-1.62) | 1.28 (1.01-1.62) | 1.26 (0.99-1.59) |
| Violence | 1.31 (1.21-1.42) | 1.31 (1.21-1.42) | **1.28 (1.18-1.39)** |
| Bullying | 1.53 (1.39-1.69) | 1.54 (1.40-1.70) | **1.51 (1.37-1.66)** |
| Community violence | 1.22 (1.11-1.33) | 1.22 (1.11-1.34) | **1.19 (1.08-1.30)** |
| Collective violence | 1.37 (1.09-1.73) | 1.37 (1.08-1.73) | **1.30 (1.03-1.65)** |
| Number of ACEs |  |  |  |
| 0 | Ref. | Ref. | Ref. |
| 1 | 1.73 (1.57-1.91) | 1.75 (1.58-1.93) | **1.72 (1.56-1.90)** |
| 2 | 1.96 (1.72-2.23) | 1.98 (1.74-2.25) | **1.96 (1.72-2.24)** |
| 3 | 2.27 (1.95-2.64) | 2.29 (1.97-2.66) | **2.23 (1.91-2.60)** |
| ≥4 | 2.83 (2.49-3.21) | 2.86 (2.52-3.25) | **2.77 (2.43-3.16)** |

**Note:** *OR*, odds ratio; *CI*, confidence interval; Model 1 was crude model; Model 2 was further adjusted for gender, race, place of residence and grade; Model 3 was additionally adjusted for sibship, family structure, maternal education, paternal education and lifestyles.

**Table S3. Associations between lifestyles and** **problematic smartphone use (PSU) among study participants (performed by multilevel model)**

| Lifestyles | *OR* (95% *CI*) | | |
| --- | --- | --- | --- |
|  | Model 1 | Model 2 | Model 3 |
| Healthy | Ref. | Ref. | Ref. |
| Intermediate | 1.55 (1.40-1.71) | 1.56 (1.41-1.72) | **1.53 (1.39-1.70)** |
| Unhealthy | 2.13 (1.86-2.43) | 2.21 (1.93-2.53) | **2.07 (1.81-2.37)** |

**Note:** *OR*, odds ratio; *CI*, confidence interval; Model 1 was crude model; Model 2 was further adjusted for gender, race, place of residence and grade; Model 3 was additionally adjusted for sibship, family structure, maternal education, paternal education and ACEs score.
